# Ethyl 4-oxo-2,3,4,9-tetra­hydro-1*H*-carbazole-3-carboxyl­ate

**DOI:** 10.1107/S1600536811018678

**Published:** 2011-05-20

**Authors:** Cevher Gündoğdu, Mustafa Göçmentürk, Yavuz Ergün, Barış Tercan, Tuncer Hökelek

**Affiliations:** a Department of Chemistry, Faculty of Arts and Sciences, Dokuz Eylül University, Tınaztepe, 35160 Buca, Izmir, Turkey; b Department of Physics, Karabük University, 78050, Karabük, Turkey; c Department of Physics, Hacettepe University, 06800 Beytepe, Ankara, Turkey

## Abstract

In the title compound, C_15_H_15_NO_3_, the carbazole skeleton includes an eth­oxy­carbonyl group at the 3-position. In the indole ring system, the benzene and pyrrole rings are nearly coplanar, forming a dihedral angle of 0.89 (4)°. The cyclo­hexenone ring has an envelope conformation. In the crystal, inter­molecular N—H⋯O and C—H⋯O hydrogen bonds link the mol­ecules into a three dimensional network. A weak C—H⋯π inter­action is also observed.

## Related literature

For background to tetra­hydro­carbazole systems present in indole-type alkaloids, see: Saxton (1983 ▶). For related structures, see: Hökelek *et al.* (1994 ▶, 1998 ▶, 1999 ▶, 2009 ▶); Patır *et al.* (1997 ▶); Hökelek & Patır (1999 ▶); Çaylak *et al.* (2007 ▶); Uludağ *et al.* (2009 ▶). For the use of 4-oxo-tetra­hydro­carbazole in the syntheses of biologically active species, see: Kumar *et al.* (2008 ▶); Ergün *et al.* (2002 ▶); Li & Vince (2006 ▶). For bond-length data, see: Allen *et al.* (1987 ▶).
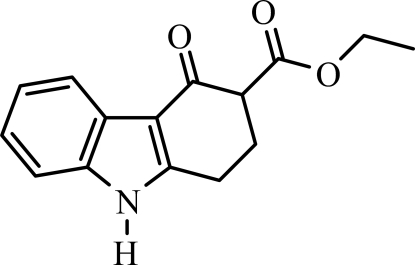


## Experimental

### 

#### Crystal data

C_15_H_15_NO_3_
*M* *_r_* = 257.28
Orthorhombic, 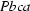

*a* = 9.1057 (3) Å
*b* = 12.7031 (4) Å
*c* = 21.3874 (5) Å
*V* = 2473.89 (13) Å^3^
*Z* = 8
Mo *K*α radiation
μ = 0.10 mm^−1^
*T* = 100 K
0.43 × 0.26 × 0.20 mm

#### Data collection

Bruker Kappa APEXII CCD diffractometer
Absorption correction: multi-scan (*SADABS*; Bruker, 2005 ▶) *T*_min_ = 0.960, *T*_max_ = 0.981
12029 measured reflections
2993 independent reflections
2258 reflections with *I* > 2σ(*I*)
*R*_int_ = 0.033

#### Refinement

*R*[*F* ^2^ > 2σ(*F* ^2^)] = 0.042
*wR*(*F* ^2^) = 0.102
*S* = 1.04
2993 reflections
177 parameters
H atoms treated by a mixture of independent and constrained refinement
Δρ_max_ = 0.28 e Å^−3^
Δρ_min_ = −0.25 e Å^−3^

### 

Data collection: *APEX2* (Bruker, 2007 ▶); cell refinement: *SAINT* (Bruker, 2007 ▶); data reduction: *SAINT*; program(s) used to solve structure: *SHELXS97* (Sheldrick, 2008 ▶); program(s) used to refine structure: *SHELXL97* (Sheldrick, 2008 ▶); molecular graphics: *ORTEP-3 for Windows* (Farrugia, 1997 ▶) and *Mercury* (Macrae *et al.*, 2006 ▶); software used to prepare material for publication: *WinGX* (Farrugia, 1999 ▶) and *PLATON* (Spek, 2009 ▶).

## Supplementary Material

Crystal structure: contains datablocks I, global. DOI: 10.1107/S1600536811018678/hb5881sup1.cif

Structure factors: contains datablocks I. DOI: 10.1107/S1600536811018678/hb5881Isup2.hkl

Supplementary material file. DOI: 10.1107/S1600536811018678/hb5881Isup3.cml

Additional supplementary materials:  crystallographic information; 3D view; checkCIF report

## Figures and Tables

**Table 1:** Hydrogen-bond geometry (Å, °) *Cg*3 is the centroid of the C5*A*/C5–C8,C8*A* ring.

*D*—H⋯*A*	*D*—H	H⋯*A*	*D*⋯*A*	*D*—H⋯*A*
N9—H9⋯O2^i^	0.885 (16)	2.044 (16)	2.9103 (15)	166.0 (15)
C3—H3⋯O1^ii^	1.00	2.41	3.4053 (17)	173
C11—H11*A*⋯*Cg*3^iii^	0.99	2.86	3.7358 (15)	148

Symmetry codes: (i) 
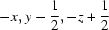
; (ii) 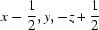; (iii) 
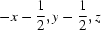
.

**Fig. 1.**
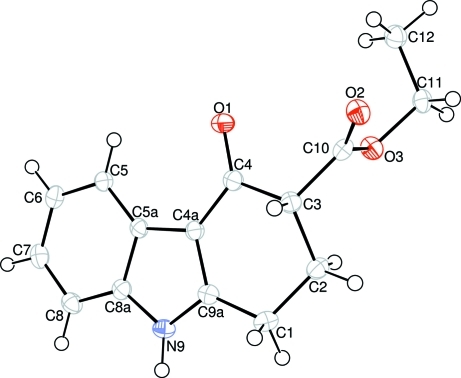
The molecular structure of (I) with displacement ellipsoids drawn at the 50% probability level.

**Fig. 2.**
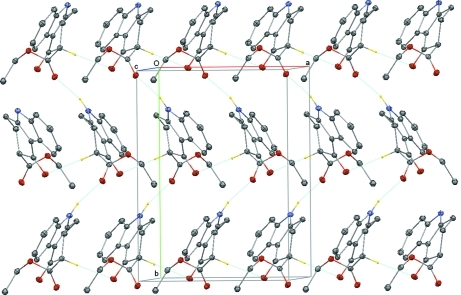
A view of the crystal packing of the title compound. The N—H···O and C—H···O hydrogen bonds are shown as dashed lines [H-atoms not involved in hydrogen bonding have been omitted for clarity].

## supplementary crystallographic information

### Comment

Tetrahydrocarbazole systems are present in the framework of a number of indole-type alkaloids of biological interest (Saxton, 1983). The structures of tricyclic, tetracyclic and pentacyclic ring systems with dithiolane and other substituents of the tetrahydrocarbazole core, have been reported previously (Hökelek *et al.*, 1994; Patır *et al.*, 1997; Hökelek *et al.*, 1998; Hökelek *et al.*, 1999; Hökelek & Patır, 1999). Although 4-oxo-tetrahydrocarbazoles rarely occur in nature, they have been increasingly important intermediates in the syntheses of indole or carbazole alkaloids and various biologically active heterocyclic compounds because of their unique structures. For instance, 4-oxo-tetrahydrocarbazole was used in the syntheses of antiemetic drugs, central nervous system active drugs and NPY-1 antagonists (Kumar *et al.*, 2008). They have also been used in the syntheses of indole alkaloids (Ergün *et al.*, 2002). Tetrahydrocarbazolone based antitumor active compounds and inhibitors of HIV integrase were synthesized from 4-oxo-tetrahydrocarbazoles (Li & Vince, 2006). The present study was undertaken to ascertain the crystal structure of the title compound, (I).

The molecule of the title compound contains a carbazole skeleton with an ethoxycarbonyl group at the 3 position, (Fig. 1), where the bond lengths are close to standard values (Allen *et al.*, 1987) and generally agree with those in the previously reported compounds. In all structures atom N9 is substituted.

An examination of the deviations from the least-squares planes through individual rings shows that rings B (C4a/C5a/C8a/N9/C9a) and C (C5a/C5—C8/C8a) are nearly coplanar [with a maximum deviation of -0.012 (1) Å for atom C5a] with dihedral angle of B/C = 0.89 (4)°. Ring A (C1—C4/C4a/C9a) adopts envelope conformation with atom C2 displaced by -0.632 (2) Å from the plane of the other rings atoms, as in 3a,4,10,10b-tetrahydro-2H-furo[2,3-a]carbazol-5(3H)-one (Çaylak *et al.*, 2007), 3,3-ethylenedithio-3,3a,4,5,10,10b-hexahydro-2H-furo[2,3-a]carbazole (Uludağ *et al.*, 2009) and ethyl 1-oxo-1,2,3,4-tetrahydro-9*H*-carbazole-3-carboxylate (Hökelek *et al.*, 2009).

In the crystal, intermolecular N—H···O and C—H···O hydrogen bonds link the molecules into a three dimensional network (Table 1 and Fig. 2). There also exists a weak C—H···π interaction (Table 1).

### Experimental

A solution of 2,3-dichloro -5,6-dicyano-p-benzoquinone (9.36 g, 41.20 mmol) in tetrahydrofuran (20 ml, 90%) was added dropwise to a solution of ethyl 2,3,4,9-tetrahydro-1*H* -carbazole-3-carboxylate (5.00 g, 20.60 mmol) in tetrahydrofuran (50 ml, 90%) at 268 K. The reaction mixture was stirred for 10 min at 268 K, and then the solution was poured into sodium hydroxide (500 ml, 10%) and extracted with ethyl acetate. The organic layer was dried with anhydrous magnesium sulfate, and the solvent was removed. The residue was purified by chromatography using silica gel and ethyl acetate. After the solvent was evaporated, the product was crystallized from ether to yield colourless blocks of (I) (yield; 0.58 g, 11%, m.p. 396 K).

### Refinement

H9 atom is located in a difference Fourier synthesis and refined isotropically. The remaining C-bound H-atoms were positioned geometrically with C—H = 0.95, 1.00, 0.99 and 0.98 Å, for aromatic, methine, methylene and methyl H-atoms, respectively, and constrained to ride on their parent atoms, with *U*_iso_(H) = k × *U*_eq_(C), where k = 1.5 for methyl H-atoms and k = 1.2 for all other H-atoms.

### Crystal data

**Table:**

C_15_H_15_NO_3_	*F*(000) = 1088
*M**_r_* = 257.28	*D*_x_ = 1.382 Mg m^−^^3^
Orthorhombic, *P**b**c**a*	Mo *K*α radiation, λ = 0.71073 Å
Hall symbol: -P 2ac 2ab	Cell parameters from 3293 reflections
*a* = 9.1057 (3) Å	θ = 2.9–28.3°
*b* = 12.7031 (4) Å	µ = 0.10 mm^−^^1^
*c* = 21.3874 (5) Å	*T* = 100 K
*V* = 2473.89 (13) Å^3^	Block, colorless
*Z* = 8	0.43 × 0.26 × 0.20 mm

### Data collection

**Table:**

Bruker Kappa APEXII CCD diffractometer	2993 independent reflections
Radiation source: fine-focus sealed tube	2258 reflections with *I* > 2σ(*I*)
graphite	*R*_int_ = 0.033
φ and ω scans	θ_max_ = 28.3°, θ_min_ = 2.9°
Absorption correction: multi-scan (*SADABS*; Bruker, 2005)	*h* = −11→9
*T*_min_ = 0.960, *T*_max_ = 0.981	*k* = −16→8
12029 measured reflections	*l* = −20→28

### Refinement

**Table:**

Refinement on *F*^2^	Primary atom site location: structure-invariant direct methods
Least-squares matrix: full	Secondary atom site location: difference Fourier map
*R*[*F*^2^ > 2σ(*F*^2^)] = 0.042	Hydrogen site location: inferred from neighbouring sites
*wR*(*F*^2^) = 0.102	H atoms treated by a mixture of independent and constrained refinement
*S* = 1.04	*w* = 1/[σ^2^(*F*_o_^2^) + (0.044*P*)^2^ + 0.6029*P*] where *P* = (*F*_o_^2^ + 2*F*_c_^2^)/3
2993 reflections	(Δ/σ)_max_ < 0.001
177 parameters	Δρ_max_ = 0.28 e Å^−^^3^
0 restraints	Δρ_min_ = −0.25 e Å^−^^3^

### Special details

**Table:**

Geometry. All esds (except the esd in the dihedral angle between two l.s. planes) are estimated using the full covariance matrix. The cell esds are taken into account individually in the estimation of esds in distances, angles and torsion angles; correlations between esds in cell parameters are only used when they are defined by crystal symmetry. An approximate (isotropic) treatment of cell esds is used for estimating esds involving l.s. planes.
Refinement. Refinement of F^2^ against ALL reflections. The weighted R-factor wR and goodness of fit S are based on F^2^, conventional R-factors R are based on F, with F set to zero for negative F^2^. The threshold expression of F^2^ > 2sigma(F^2^) is used only for calculating R-factors(gt) etc. and is not relevant to the choice of reflections for refinement. R-factors based on F^2^ are statistically about twice as large as those based on F, and R- factors based on ALL data will be even larger.

### Fractional atomic coordinates and isotropic or equivalent isotropic displacement parameters (Å^2^)

**Table:**

	*x*	*y*	*z*	*U*_iso_*/*U*_eq_	
O1	0.27325 (11)	0.49989 (7)	0.23207 (4)	0.0234 (2)	
O2	0.13644 (11)	0.53475 (7)	0.37765 (4)	0.0226 (2)	
O3	0.31033 (11)	0.40993 (7)	0.36958 (4)	0.0220 (2)	
C1	0.01764 (17)	0.22360 (9)	0.25890 (6)	0.0217 (3)	
H1A	0.0260	0.1466	0.2651	0.026*	
H1B	−0.0879	0.2422	0.2578	0.026*	
C2	0.09311 (17)	0.28148 (9)	0.31263 (7)	0.0218 (3)	
H2A	0.1906	0.2495	0.3202	0.026*	
H2B	0.0340	0.2730	0.3511	0.026*	
C3	0.11251 (16)	0.39973 (9)	0.29874 (6)	0.0196 (3)	
H3	0.0122	0.4306	0.2936	0.024*	
C4	0.19696 (16)	0.42026 (9)	0.23804 (6)	0.0191 (3)	
C4A	0.17494 (15)	0.34286 (9)	0.18970 (6)	0.0178 (3)	
C5	0.30954 (16)	0.40632 (10)	0.08810 (6)	0.0200 (3)	
H5	0.3511	0.4689	0.1048	0.024*	
C5A	0.22407 (15)	0.34016 (9)	0.12551 (6)	0.0176 (3)	
C6	0.33239 (17)	0.37872 (11)	0.02632 (7)	0.0231 (3)	
H6	0.3903	0.4232	0.0005	0.028*	
C7	0.27209 (17)	0.28667 (10)	0.00100 (7)	0.0244 (3)	
H7	0.2901	0.2697	−0.0416	0.029*	
C8	0.18676 (17)	0.22010 (10)	0.03698 (7)	0.0229 (3)	
H8	0.1457	0.1576	0.0200	0.027*	
C8A	0.16323 (15)	0.24812 (10)	0.09904 (7)	0.0192 (3)	
N9	0.08119 (13)	0.19804 (9)	0.14526 (5)	0.0202 (3)	
H9	0.0272 (18)	0.1408 (13)	0.1399 (7)	0.029 (4)*	
C9A	0.08866 (15)	0.25402 (9)	0.19910 (6)	0.0181 (3)	
C10	0.18566 (16)	0.45682 (10)	0.35239 (6)	0.0187 (3)	
C11	0.39176 (16)	0.45682 (10)	0.42153 (7)	0.0212 (3)	
H11A	0.4609	0.4042	0.4388	0.025*	
H11B	0.3222	0.4767	0.4551	0.025*	
C12	0.47603 (17)	0.55264 (10)	0.40117 (7)	0.0246 (3)	
H12A	0.5356	0.5785	0.4361	0.037*	
H12B	0.4071	0.6076	0.3881	0.037*	
H12C	0.5403	0.5342	0.3661	0.037*	

### Atomic displacement parameters (Å^2^)

**Table:**

	*U*^11^	*U*^22^	*U*^33^	*U*^12^	*U*^13^	*U*^23^
O1	0.0268 (6)	0.0194 (4)	0.0241 (6)	−0.0053 (4)	0.0006 (5)	−0.0008 (4)
O2	0.0235 (6)	0.0223 (4)	0.0220 (6)	0.0049 (4)	−0.0009 (4)	−0.0027 (4)
O3	0.0217 (6)	0.0210 (4)	0.0233 (5)	0.0045 (4)	−0.0023 (4)	−0.0024 (4)
C1	0.0210 (8)	0.0174 (6)	0.0265 (8)	−0.0011 (5)	0.0036 (6)	−0.0004 (5)
C2	0.0251 (8)	0.0185 (6)	0.0216 (8)	−0.0003 (5)	0.0050 (6)	0.0007 (5)
C3	0.0213 (8)	0.0171 (6)	0.0204 (7)	0.0022 (5)	−0.0005 (6)	−0.0008 (5)
C4	0.0188 (8)	0.0172 (6)	0.0212 (8)	0.0027 (5)	−0.0041 (6)	0.0012 (5)
C4A	0.0171 (8)	0.0177 (6)	0.0186 (7)	0.0003 (5)	−0.0022 (6)	0.0015 (5)
C5	0.0199 (8)	0.0201 (6)	0.0199 (8)	−0.0003 (5)	−0.0031 (6)	0.0019 (5)
C5A	0.0171 (8)	0.0172 (6)	0.0184 (7)	0.0030 (5)	−0.0034 (6)	0.0005 (5)
C6	0.0227 (8)	0.0261 (7)	0.0206 (8)	0.0027 (6)	−0.0006 (6)	0.0047 (5)
C7	0.0268 (9)	0.0283 (7)	0.0179 (7)	0.0057 (6)	−0.0011 (6)	−0.0012 (6)
C8	0.0245 (8)	0.0210 (6)	0.0231 (8)	0.0026 (5)	−0.0036 (6)	−0.0036 (5)
C8A	0.0183 (8)	0.0184 (6)	0.0207 (8)	0.0031 (5)	−0.0017 (6)	0.0009 (5)
N9	0.0205 (7)	0.0164 (5)	0.0236 (7)	−0.0011 (5)	0.0009 (5)	−0.0028 (4)
C9A	0.0157 (7)	0.0165 (5)	0.0222 (7)	0.0029 (5)	−0.0014 (6)	−0.0007 (5)
C10	0.0187 (8)	0.0185 (6)	0.0190 (7)	0.0005 (5)	0.0026 (6)	0.0040 (5)
C11	0.0218 (8)	0.0241 (6)	0.0178 (7)	0.0035 (5)	−0.0026 (6)	0.0012 (5)
C12	0.0246 (9)	0.0249 (6)	0.0243 (8)	0.0008 (6)	−0.0021 (7)	0.0003 (5)

### Geometric parameters (Å, °)

**Table:**

O1—C4	1.2338 (15)	C5A—C5	1.3972 (19)
O2—C10	1.2136 (15)	C5A—C8A	1.4123 (17)
O3—C10	1.3336 (17)	C6—C7	1.4008 (19)
O3—C11	1.4626 (16)	C6—H6	0.9500
C1—C9A	1.4844 (19)	C7—C8	1.382 (2)
C1—H1A	0.9900	C7—H7	0.9500
C1—H1B	0.9900	C8—H8	0.9500
C2—C1	1.5276 (19)	C8A—C8	1.391 (2)
C2—H2A	0.9900	N9—C8A	1.3928 (18)
C2—H2B	0.9900	N9—C9A	1.3550 (17)
C3—C2	1.5413 (17)	N9—H9	0.886 (17)
C3—C4	1.531 (2)	C10—C3	1.5121 (19)
C3—H3	1.0000	C11—H11A	0.9900
C4—C4A	1.4409 (18)	C11—H11B	0.9900
C4A—C5A	1.4442 (19)	C12—C11	1.5033 (19)
C4A—C9A	1.3897 (17)	C12—H12A	0.9800
C5—C6	1.383 (2)	C12—H12B	0.9800
C5—H5	0.9500	C12—H12C	0.9800
			
C10—O3—C11	117.33 (10)	C5—C6—H6	119.3
C2—C1—H1A	109.9	C7—C6—H6	119.3
C2—C1—H1B	109.9	C6—C7—H7	119.5
C9A—C1—C2	109.08 (11)	C8—C7—C6	121.04 (14)
C9A—C1—H1A	109.9	C8—C7—H7	119.5
C9A—C1—H1B	109.9	C7—C8—C8A	117.48 (13)
H1A—C1—H1B	108.3	C7—C8—H8	121.3
C1—C2—C3	112.07 (11)	C8A—C8—H8	121.3
C1—C2—H2A	109.2	N9—C8A—C5A	107.69 (12)
C1—C2—H2B	109.2	C8—C8A—N9	130.03 (12)
C3—C2—H2A	109.2	C8—C8A—C5A	122.28 (13)
C3—C2—H2B	109.2	C8A—N9—H9	125.5 (10)
H2A—C2—H2B	107.9	C9A—N9—C8A	109.67 (11)
C2—C3—H3	107.4	C9A—N9—H9	124.7 (10)
C4—C3—C2	112.77 (11)	N9—C9A—C1	125.01 (12)
C4—C3—H3	107.4	N9—C9A—C4A	109.36 (12)
C10—C3—C2	111.82 (11)	C4A—C9A—C1	125.62 (12)
C10—C3—C4	109.90 (11)	O2—C10—O3	123.78 (13)
C10—C3—H3	107.4	O2—C10—C3	124.50 (13)
O1—C4—C3	120.68 (12)	O3—C10—C3	111.71 (11)
O1—C4—C4A	124.31 (13)	O3—C11—C12	111.62 (11)
C4A—C4—C3	114.98 (11)	O3—C11—H11A	109.3
C4—C4A—C5A	130.93 (12)	O3—C11—H11B	109.3
C9A—C4A—C4	121.94 (12)	C12—C11—H11A	109.3
C9A—C4A—C5A	107.06 (11)	C12—C11—H11B	109.3
C5A—C5—H5	120.7	H11A—C11—H11B	108.0
C6—C5—C5A	118.59 (13)	C11—C12—H12A	109.5
C6—C5—H5	120.7	C11—C12—H12B	109.5
C5—C5A—C4A	134.64 (12)	C11—C12—H12C	109.5
C5—C5A—C8A	119.13 (12)	H12A—C12—H12B	109.5
C8A—C5A—C4A	106.21 (11)	H12A—C12—H12C	109.5
C5—C6—C7	121.47 (14)	H12B—C12—H12C	109.5
			
C10—O3—C11—C12	−77.69 (15)	C5A—C4A—C9A—C1	178.62 (12)
C11—O3—C10—O2	−0.64 (19)	C5A—C4A—C9A—N9	−0.36 (15)
C11—O3—C10—C3	−179.50 (10)	C5A—C5—C6—C7	−0.1 (2)
C2—C1—C9A—N9	159.90 (13)	C4A—C5A—C5—C6	−178.78 (14)
C2—C1—C9A—C4A	−18.93 (18)	C8A—C5A—C5—C6	−0.4 (2)
C3—C2—C1—C9A	47.43 (15)	C4A—C5A—C8A—N9	0.09 (14)
C4—C3—C2—C1	−56.21 (16)	C4A—C5A—C8A—C8	179.53 (13)
C10—C3—C2—C1	179.34 (12)	C5—C5A—C8A—N9	−178.68 (12)
C2—C3—C4—O1	−149.89 (13)	C5—C5A—C8A—C8	0.8 (2)
C2—C3—C4—C4A	32.36 (16)	C5—C6—C7—C8	0.3 (2)
C10—C3—C4—O1	−24.40 (17)	C6—C7—C8—C8A	0.0 (2)
C10—C3—C4—C4A	157.85 (11)	N9—C8A—C8—C7	178.76 (13)
O1—C4—C4A—C5A	−3.9 (2)	C5A—C8A—C8—C7	−0.5 (2)
O1—C4—C4A—C9A	179.48 (13)	C8A—N9—C9A—C1	−178.57 (12)
C3—C4—C4A—C5A	173.73 (13)	C8A—N9—C9A—C4A	0.42 (15)
C3—C4—C4A—C9A	−2.87 (18)	C9A—N9—C8A—C5A	−0.31 (15)
C4—C4A—C5A—C8A	−176.82 (14)	C9A—N9—C8A—C8	−179.70 (14)
C4—C4A—C5A—C5	1.7 (3)	O2—C10—C3—C2	−126.61 (14)
C9A—C4A—C5A—C5	178.65 (15)	O2—C10—C3—C4	107.35 (15)
C9A—C4A—C5A—C8A	0.16 (14)	O3—C10—C3—C2	52.23 (15)
C4—C4A—C9A—N9	176.95 (12)	O3—C10—C3—C4	−73.80 (13)
C4—C4A—C9A—C1	−4.1 (2)		

### Hydrogen-bond geometry (Å, °)

**Table:**

Cg3 is the centroid of the C5A/C5–C8,C8A ring.

**Table:**

*D*—H···*A*	*D*—H	H···*A*	*D*···*A*	*D*—H···*A*
N9—H9···O2^i^	0.885 (16)	2.044 (16)	2.9103 (15)	166.0 (15)
C3—H3···O1^ii^	1.00	2.41	3.4053 (17)	173
C11—H11A···Cg3^iii^	0.99	2.86	3.7358 (15)	148

Symmetry codes: (i) −*x*, *y*−1/2, −*z*+1/2; (ii) *x*−1/2, *y*, −*z*+1/2; (iii) −*x*−1/2, *y*−1/2, *z*.
